# Lipid-altering effects of a dietary supplement tablet containing free plant sterols and stanols in men and women with primary hypercholesterolaemia: a randomized, placebo-controlled crossover trial

**DOI:** 10.3109/09637486.2011.636345

**Published:** 2012-06

**Authors:** Kevin C Maki, Andrea L Lawless, Matthew S Reeves, Mary R Dicklin, Belinda H Jenks, ED Shneyvas, James R Brooks

**Affiliations:** 1 Provident Clinical Research/Biofortis North America, 489 Taft Avenue, Glen Ellyn, IL 60137, USA; 2 Provident Clinical ResearchlBiofortis North America, Addison, IL, USA; 3 Pharmavite, LLC, Northridge, CA, USA

**Keywords:** phytosterols, dietary supplements, low-density lipoprotein cholesterol, non-high-density lipoprotein cholesterol

## Abstract

This randomized, placebo-controlled, crossover trial assessed the lipid-altering efficacy of a dietary supplement (tablet form) providing 1.8g/day free (non-esterified) plant sterols and stanols versus placebo for 6 weeks as part of a therapeutic lifestyle changes (TLC) diet in 32 men and women with primary hypercholesterolaemia. Mean ± SE baseline (end of a 5-week TLC diet lead-in) lipid concentrations (mmol/1) were total cholesterol (TC), 5.88 ± 0.08; non-high-density lipoprotein cholesterol (non-HDL-C), 4.71 ± 0.09; low-density lipoprotein cholesterol (LDL-C), 4.02 ± 0.08; HDL-C, 1.17 ± 0.06 and triglycerides (TGs), 1.51 ± 0.12. Differences from control in responses (plant sterol/stanol — control) were significant (*p* < 0.05) for LDL-C (− 4.9%), non-HDL-C (− 3.6%) and TC (− 2.8%). HDL-C and TG responses were not significantly different between treatment conditions. These results indicate that 1.8g/day free plant sterols/stanols administered in a tablet produced favourable lipoprotein lipid changes in men and women with hypercholesterolaemia.

## Introduction

The guidelines of the National Cholesterol Education Program (NCEP) recommend incorporation of 2g/day of plant sterols or stanols (phytosterols or phytostanols) into the therapeutic lifestyle changes (TLC) diet as a dietary adjunct for individuals who do not achieve their low-density lipoprotein cholesterol (LDL-C) treatment targets with diet alone (NCEP Expert Panel 2002). A large body of evidence supports the efficacy of plant sterol/stanol-enriched products providing 1.0–3.0 g/day plant sterols/stanols for lowering LDL-C, non-high-density lipoprotein (HDL)-C and total cholesterol (TC) concentrations (Food and Drug Administration 2000; NCEP Expert Panel 2002; Maki et al. 2003; Demonty et al. 2009; Sánchez-Muniz et al. 2009). Results from studies in subjects with hypercholesterolaemia have generally indicated that HDL-C and triglyceride (TG) concentrations are unchanged by consumption of plant sterols or stanols, although some evidence suggests that plant stanols (and presumably sterols) may lower the TG concentration in subjects with hypertriglycer-idemia (Plat et al. 2009).

Because of their structural similarity to cholesterol, plant sterols and stanols compete with cholesterol for incorporation into micelles, as well as for transport across the brush border by the Nieman Pick C1 - Like 1 transporter (von Bergmann et al. 2005; Hui et al. 2008; Jones 2008). In addition, the accumulation of plant sterols or stanols in the enterocyte appears to trigger the production of adenosine triphosphate binding cassette transporters G5 and G8, which function to transport sterols, including cholesterol, out of the enterocyte, into the intestinal lumen (von Bergmann et al. 2005; Hui et al. 2008; Jones 2008). The net result of these mechanisms is to reduce intestinal cholesterol absorption (Ikeda and Sugano 1998; Jones 2008). Consequently, hepatic cholesterol content is reduced, inducing an up-regulation of LDL receptor expression, thereby enhancing the removal of LDL and other apolipoprotein B-containing lipoprotein particles from the circulation (Jones 2008).

Various commercially available products contain plant sterols and/or stanols in free and/or esterified forms including margarine-type spreads, yogurt and yogurt-based drinks, orange juice and dietary supplements in capsule or tablet form. Most clinical trials examining the effects of plant sterols/stanols on lipid concentrations have administered food forms (Ostlund et al. 1999; Maki et al. 2001, 2003; Nestel et al. 2001; Volpe et al. 2001; de Graaf et al. 2002; Gremaud et al. 2002; Pouteau et al. 2003; Berger et al. 2004; Thomsen et al. 2004; Doornbos et al. 2006; Abumweis et al. 2008), while fewer have investigated the effects of tablet and capsule forms (McPherson et al. 2005; Goldberg et al. 2006; Acuff et al. 2007; Carr et al. 2009). The use of plant sterol/stanol capsules or tablets offers a practical option compared with traditional food applications because it provides a vehicle that can be easily incorporated into a cholesterol-lowering regimen without impacting dietary macronutrient distribution.

Recently, the US Food and Drug Administration reviewed the published data on phytosterols and risk for coronary heart disease (CHD; Food and Drug Administration 2010). In their review they concluded that ‘the available scientific evidence for the cholesterol-lowering effects of phytosterols in dietary supplements shows that formulation of the supplement product is an important factor in the effectiveness of the product’. They further concluded that ‘the totality of available scientific evidence for the cholesterol-lowering effects of nonesterified phytosterols in dietary supplements is inconsistent’. The agency requested submission of additional data to demonstrate the cholesterol-lowering efficacy of nonesterified phytosterols consumed as ingredients in dietary supplements. This trial was undertaken to assess the efficacy of a dietary supplement in a tablet providing a total of 1.8g/day plant sterol/stanol in a non-esterified form, as part of a NCEP TLC diet, for improving the lipoprotein lipid profile in men and women with primary hypercholesterolaemia. Similar doses of non-esterified plant sterol/stanol administered in food products have been shown to significantly lower LDL-C levels (Jones et al. 1999; de Graaf et al. 2002). Daily plant sterol intake in the US diet is ∼200mg (range 150–450 mg in various Western populations; Ostlund 2002), which is insufficient to materially alter cholesterol levels. Accordingly, the dosage of 1.8 g was chosen with the expectation that total plant sterol/stanol intake in study subjects would be ∼2.0g/day from a combination of diet and the supplement provided. A dose of 1.7g/day non-esterified sitostanol-containing phytosterols administered in margarine for 30 days resulted in a 15.5% LDL-C reduction (Jones et al. 1999), and 1.8 g/d administered in chocolate for 4 weeks decreased LDL-C by 10.3% (de Graaf et al. 2002).

## Methods

### Study design

This was a randomized crossover study consisting of a 5-week diet plus single-blind placebo lead-in, followed by two double-blind 6-week treatment periods during which subjects received either dietary supplement tablets (four tablets daily, two tablets with each of two meals) providing 1.8g/day non-esterified plant sterols/stanols or the same number of matching placebo tablets. The study was conducted at two clinical research centres (Provident Clinical Research in Addison, IL, USA, and Bloomington, IN, USA) according to Good Clinical Practice Guidelines, the Declaration of Helsinki and the US 21 Code of Federal Regulations. Informed consent for the study was obtained from all subjects before protocol-specific procedures were carried out and subjects were informed of their rights to withdraw from the study at any time.

### Subjects

Men and women of age 21–79 years, inclusive, each with a fasting LDL-C level ≥3.4 and <5.7mmol/l, and in good general health on the basis of medical history and routine laboratory tests were eligible for the study. Individuals were excluded from participation if they had a body mass index of > 42.0 kg/m^2^, fasting blood glucose ≥7.0 mmol/l or diabetes mellitus, resting blood pressure ≥ 160 mm Hg systolic and/or ≥ 100 mm Hg diastolic, or CHD or a CHD risk equivalent as defined by the NCEP Adult Treatment Panel III (NCEP Expert Panel 2002). Additional exclusion criteria included a history of extreme dietary habits, eating disorders, alcoholism, cancer or any clinically important cardiovascular disorders. Use of any medications, dietary supplements or fortified foods with lipid-altering effects, including sterol and stanol products, were excluded for at least 4 weeks prior to study entry as was the use of weight-loss drugs or programmes and recent body weight change > 4.5 kg.

### Study products and diet instruction

Following the diet and the single-blind placebo lead-in, subjects were randomly assigned to receive, in a double-blind manner, either active (non-esterified sterol/stanol tablets, 0.45 g per tablet) or control (matched placebo tablets, the main ingredient of which was lactose) study product for 6 weeks (treatment period I) and then cross over to receive the opposite product for 6 weeks (treatment period II). Subjects were instructed to swallow, with water or another beverage, four active or control tablets daily (two with each of two meals) at consistent times each day. Compliance with active and control tablets was assessed by counting unused study product returned to the clinic. Subjects were instructed to maintain their usual physical activity patterns throughout the trial.

Beginning at week –5 and throughout the study, subjects were counselled to follow the weight maintenance version of the TLC diet recommended by the NCEP, and handouts were provided to reinforce the diet instructions (NCEP Expert Panel 2002). To evaluate compliance with the TLC diet, diet records were completed on 3 consecutive days (2 weekdays and 1 weekend day) at baseline and the end of each treatment period using standardized forms that included instructions for obtaining complete information such as the use of condiments, brand names and cooking methods. Daily intakes of energy and key nutrients were calculated from these records utilizing Food Processor SQL Software (version 10.4, EHSA Research, Salem, OR, USA). Body weight was measured at each clinic visit throughout the trial.

### Laboratory measurements

Fasting (9–15h) plasma lipid profile measurements from blood samples collected in duplicate at baseline (weeks – 1 and 0) and at the end of each treatment period (weeks 5 and 6, 11 and 12) were conducted by the EMH Reference Laboratory (Elmhurst, IL, USA) according to the Standardization Program of the Centers for Disease Control and Prevention and the National Heart, Lung, and Blood Institute. Lipoprotein lipid assessments included TC, non-HDL-C (calculated as TC-HDL-C), LDL-C, HDL-C and TG in mg/dl and converted to mmol/1 with conversion factors of 0.02586 for cholesterol and 0.01129 for TG. LDL-C concentration was calculated using Friedewald's (1972) equation as follows: LDL-C = TC − HDL-C − TG/5. Because this equation is not valid when the TG concentration is above 4.51 mmol/1, LDL-C values were not calculated in the few instances when subjects had values in this range.

### Statistical analyses

Statistical analyses were completed in SAS version 9.2 (SAS Institute, Cary, NC, USA). Efficacy analyses were performed on the sample that included all subjects who were randomized and provided at least one post-randomization fasting lipid profile during each treatment condition. The sample size for this study was selected for 90% power (5% α-level) to detect a 7% difference between treatments in the change from baseline LDL-C concentration. Baseline characteristics for subjects in the two treatment sequences were compared using analysis of variance (continuous variables) or Fisher's exact test (categorical variables). Repeated measures analysis of covariance (using SAS PROC MIXED) was used to compare lipid, dietary intake and vital signs response variables (changes or percent changes from baseline) for the two treatment conditions (active and control) using the baseline value as a covariate. The initial model included subject as a random effect and terms for treatment condition, sequence and treatment by sequence interaction. If the treatment by sequence interaction term for a variable was not statistically significant (*p* > 0.05), it was dropped from the final model.

Examination of responses by sequence suggested that no material differences were present that would bring into question the appropriateness of pooling data from the two sequence groups. Residuals from the final model were examined to assess normality, and if clear evidence of non-normality was present, rank transformations were employed in the final models. An additional analysis of covariance was conducted using the results of Kris-Etherton and Yu's (1997) predictive equation for LDL-C changes with response to dietary alteration with and without an adjustment for dietary cholesterol intake based on Hegsted et al.'s (1993) equation according to diet record results. This was done in order to assess possible confounding by dietary changes during the treatment periods. Safety was assessed by the evaluation of treatment emergent adverse events and changes in vital signs measurements. McNemar's test was used for statistical comparisons between treatment conditions for categorical variables.

## Results

### Subjects and demographics

**Figure 1:**
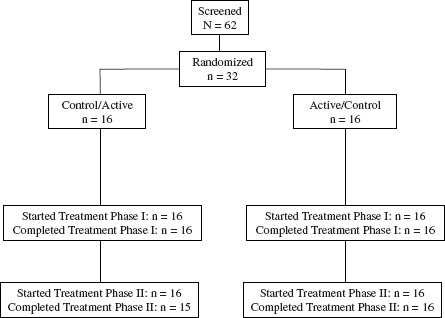
Participant flow through the study.

Sixty-two subjects were screened, 32 of whom entered and completed the first treatment period (Figure 1). One subject withdrew consent after completing part of the second treatment period while receiving the active study product, but was retained in the efficacy analyses. Demographic and baseline characteristics of the subjects are presented in Table I. Subjects were predominantly female (59%), of non-Hispanic White race/ethnicity (91%) and non-smokers (91%). Participants had a mean age of 57.6 years and a mean body mass index of 27.4 kg/m^2^. Compliance with active and control tablets was 98.0% and 98.4% of the expected tablets, respectively (*p* = 0.586).

**Table I:** Subject characteristics at baseline.*

Characteristic	Total *N* = 32
	*n* (%)
Male	13 (41)
Female	19 (59)
Race/ethnicity	
Non-HispanicWhite	29 (91)
Asian	2(6)
Black/African-American	1(3)
Smoking status	
Non-smoker	29 (91)
Current smoker	3(9)
	Mean ± SEM
Age (years)	57.6 ± 2.1
Weight (kg)	77.4 ± 2.5
Body mass index (kg/m^2^)	27.4 ± 0.7
Systolic blood pressure (mm Hg)	119.5 ± 1.9
Diastolic blood pressure (mm Hg)	73.7 ± 1.5
Fasting glucose (mmol/l)†	5.23 ± 0.11

Note: SEM, standard error of the mean;

* Results for both treatment sequences were pooled;

† To convert glucose from mmol/l to mg/dl, multiply by 18.

### Lipids

Fasting plasma lipids at baseline, percent changes from baseline for control and active treatments and differences in responses between conditions are shown in Table II. Differences from control in responses (plant sterol/stanol — control) were significant (*p* <0.05) for LDL-C (−4.9%), non-HDL-C (−3.6%) and TC (−2.8%). HDL-C and TG responses were not significantly different between treatment conditions.

**Table II:** Fasting lipids at baseline, percent changes from baseline and differences in responses between treatments. *,†

Parameter	Baseline (mmol/l)‡	Control (%Δ)	Active(%Δ)	% difference in response	*p*-Value
Mean (SEM) or mean					
LDL-C	4.02 (0.08)	0.6 (1.4)	−4.3 (1.9)	−4.9	0.002
Non-HDL-C	4.71 (0.09)	−1.4 (1.2)	−5.0 (1.5)	−3.6	0.008
TC	5.88 (0.08)	−0.5 (1.1)	−3.3 (1.3)	−2.8	0.024
HDL-C	1.17 (0.06)	3.8 (1.8)	4.1 (2.4)	0.3	0.889
TG	1.51 (0.12)	−8.4 (4.1)	−4.2 (4.6)	4.2	0.281

Notes: HDL-C, high-density lipoprotein cholesterol;LDL-C, low-density lipoprotein cholesterol; SEM, standard error of the mean; TC, total cholesterol; TG, triglycerides;

* Results for both treatment sequences were pooled. Adjusting for differences in dietary in take did not significantly alter results;

† Baseline = average of values at weeks − 1and 0; control and active, % Δ = percentage change from baseline to the average of values at the last 2 weeks of each treatment period (weeks 5 and 6, weeks 11and 12); difference in response = active % change − control % change;

‡ To convert from mmol/l to mg/dl, for cholesterol multiply by 38.7 and for TG multiply by 88.6.

### Diet

Total reported energy intake at baseline was 1634 kcal/day and increased by 142.5 kcal/day during the active condition compared with a decrease of 8.4 kcal/day during the control condition, a difference which approached statistical significance (*p* = 0.056). There were no significant differences in changes from baseline between active and control conditions, respectively, in percentages of intake from carbohydrate (−1.1% and 1.5%, *p* = 0.100), protein (−0.3% and 0.3%, *p* = 0.513), total fat (0.3% and − 1.7%, *p* = 0.128) and saturated fat (1.1% and 0.2%, *p* = 0.199); and intakes of dietary fibre (0.4 and 0.9 g/day, *p* = 0.645), soluble fibre (0.05 and −0.01 g/d, *p* = 0.376) or cholesterol (30.8 and 29.5 mg/day, *p* = 0.762). Analysis of covariance to adjust for differences in energy intake or predicted LDL-C response using prediction equations (as described in ‘Statistical analysis’ section) did not materially alter the estimates of the treatment effects (data not shown).

### Vital signs, body weight and safety

There were no statistically significant or clinically relevant changes in vital signs, body weight or clinical laboratory values. Mean body weight changed < 0.5 kg and mean systolic and diastolic blood pressures changed ≤ 1 mm Hg throughout the study. Adverse events assessed by non-leading questions at each clinic visit were reported by eight (12.5%) of the subjects during the active period and seven (10.9%) of the subjects during the control period. The most common adverse events were related to the respiratory system (rhinitis and sinusitis). None of the adverse events were serious and all but one adverse event (moderate endometrial hyperplasia experienced by one subject when receiving the placebo) were reported to be mild. Increased appetite reported by one subject during the control period was considered by the study physician to be possibly related to study product. All other adverse events were classified by the study physicians as not related or unlikely to be related to the study products.

## Discussion

The results of the present study indicate that 1.8 g/day unesterified plant sterols/stanols administered orally in tablets resulted in significant reductions in atherogenic lipoprotein lipids (LDL-C, non-HDL-C and TC) in individuals with hypercholesterolaemia. The LDL-C lowering of 4.9% was within the expected range, albeit at the lower end, based on meta-analyses (Katan et al. 2003; Abumweis et al. 2008; Demonty et al. 2009). According to calculations from a meta-analysis, weighted mean percentage reductions in LDL-C range from ∼5% at 1.0 g/day phytosterol to ∼11% at 3.0 g/day phytosterol with no appreciable increase in response at higher dosages up to 9.0 g/day (Demonty et al. 2009).

Although LDL-C has long been considered the principal lipoprotein determinant of atherosclerosis, results from observational studies and clinical trials of lipid-altering therapies have consistently shown that the non-HDL-C level is a better predictor of CHD event risk than LDL-C alone, irrespective of whether the TG concentration is elevated (Cui et al. 2001; Bittner 2003; Pischon et al. 2005; Ridker et al. 2005; Liu et al. 2006; Miller et al. 2008). Non-HDL-C was reduced by 3.6% in the present trial.

Most prior clinical trials have been conducted using plant sterols and stanols provided in foods (Ostlund et al. 1999; Maki et al. 2001, 2003; Nestel et al. 2001; Volpe et al. 2001; de Graaf et al. 2002; Gremaud et al. 2002; Pouteau et al. 2003; Berger et al. 2004; Thomsen et al. 2004; Doornbos et al. 2006; Abumweis et al. 2008). Relatively few studies have investigated plant sterols/stanols in tablet or capsule forms, but the available results support the ability of plant sterols and stanols to reduce LDL-C concentrations in some supplement forms (McPherson et al. 2005; Goldberg et al. 2006; Acuff et al. 2007; Carr et al. 2009). Goldberg et al. (2006) demonstrated that tablets providing 1.8 g/day soy stanols in addition to the subjects’ usual statin therapy, reduced LDL-C by 9.1 %. McPherson et al. (2005) reported a reduction in LDL-C of 10.4% after administration of stanol lecithin tablets providing 1.26 g stanols/day. The use of plant sterols and stanols in capsules or tablets offers a practical option compared to traditional phytosterol-containing food vehicles by virtue of being easily incorporated into a cholesterol-lowering regimen without altering the macronutrient distribution. This feature might increase compliance during long-term use compared with the types of dietary changes needed to incorporate phytosterol-containing foods (Berger et al. 2004).

Because of the hypothesized mechanisms of action: (1) competing with cholesterol for incorporation into micelles and (2) up-regulation of the production of sterol transport proteins from the enterocyte into the intestinal lumen, it is recommended that sterol or stanol products be consumed with meals to ensure adequate availability of bile acids for micelle formation (Berger et al. 2004). In the present study, all subjects were advised to take the tablets twice daily with meals. It was believed at one time that plant sterols would be more effective when consumed with diets containing higher levels of cholesterol or fat (Mussner et al. 2002; Berger et al. 2004). Since only 10–20% of cholesterol passing through the intestine daily is of dietary origin, sterols and stanols appear to be equally effective when consumed with low-fat diets such as the weight maintenance version of the TLC diet used in the current study (Hallikainen and Uusitupa 1999; Jones et al. 1999; Hallikainen et al. 2000; Katan et al. 2003; Abumweis et al. 2008; Demonty et al. 2009).

Additional research is needed to further evaluate the effects of this and other plant sterol/stanol products on lipid levels in other types of dyslipidemia, particularly Fredrickson Type IIb (mixed) dyslipidemia. Also of interest would be an evaluation of the effect on cholesterol biosynthesis of the intake of plant sterol/stanol tablets during the day (only with lunch) versus in the evening (only with dinner).

Intervention trials have shown that each 1% reduction in LDL-C (or non-HDL-C) lowers the risk of a major cardiovascular event by ∼ 1% over a period of 5 years (Grundy et al. 2004; Robinson et al. 2005, 2009). However, the cardiovascular benefit of maintaining low levels of atherogenic lipoprotein cholesterol levels over decades may be larger than would be predicted on the basis of results from short-term cholesterol-lowering intervention trials. Each 1% reduction in LDL-C or non-HDL-C may be associated with as much as a 3% reduction in CHD event risk if maintained over an extended period (Cohen et al. 2006). Thus, the changes in atherogenic lipoprotein cholesterol observed in the present study are clinically relevant.

## Conclusions

In conclusion, incorporation of a dietary supplement tablet containing 1.8 g/day unesterified plant sterols/stanols into the NCEP TLC diet produced favourable changes in apolipoprotein B-containing lipoprotein lipids in individuals with hypercholesterolaemia and would be expected to reduce the risk for cardiovascular disease if consumed over an extended period of time (Miettinen and Gylling 2004).
